# Keep calm and keep rowing: the psychophysical effects of dragon boat program in breast cancer survivors

**DOI:** 10.1007/s00520-024-08420-7

**Published:** 2024-03-08

**Authors:** Tatiana Moro, Andrea Casolo, Veronica Bordignon, Alessandro Sampieri, Giorgia Schiavinotto, Lisa Vigo, Marta Ghisi, Antonio Paoli, Silvia Cerea

**Affiliations:** 1 Department of Biomedical Sciences, Istituto Di Fisiologia, University of Padova, Via Marzolo 3, 35131 Padua, Italy; 2 Department of General Psychology, University of Padova, Padua, Italy; 3 Unità Operativa Complessa (UOC) Hospital Psychology, University-Hospital of Padova, Padua, Italy

**Keywords:** Dragon Boat, Breast cancer, Motor skills, Body image, Quality of life

## Abstract

**Purpose:**

Dragon Boat discipline has become a popular type of physical exercise among women with breast cancer. The present study aims to investigate the effects of Dragon Boat activity on body composition, physical function, and psychosocial aspects (i.e., body appreciation and quality of life [QoL]) in women operated for breast cancer.

**Methods:**

Thirty-one women (age, 57.88 ± 7.88 years; BMI, 27.86 ± 6.38 kg·m^−2^) with a previous breast removal surgery were recruited and randomized into two groups: Dragon Boat group (DB, *N* = 18) or a home-based non-supervised training program (home exercise group; HG, *N* = 13). All participants underwent body composition, handgrip test, 30-s chair stand test (30CST), 6-min walking test (6MWT), and shoulder mobility measurements at baseline and after 12 weeks of intervention. Participants also filled out the Body Appreciation Scale-2 (BAS-2) and the Short Form Health Survey-12 (SF-12) self-report questionnaires.

**Results:**

Dragon Boat activity significantly improved the 30CST (+ 6%, *p* = .011) and 6MWT performance (+ 30%, *p* = .011) compared to a home-based non-supervised training program. Moreover, 20% (3/15 women) of women in the DB group obtained a reliable change from pre- to post-intervention in the BAS-2 and in the mental QoL component of the SF-12 (*vs* 15% and 0% of the HC group). No reliable change emerged for the physical component of the SF-12.

**Conclusion:**

Dragon Boat activity is efficient to improve lower limb strength in women operated for breast cancer. Furthermore, Dragon Boat activity emerged to improve body appreciation and mental QoL in some of the women assigned to this activity. Importantly, no adverse events were documented during the intervention.

**Trial registration:**

NCT05206526 (10/02/2022)

**Supplementary Information:**

The online version contains supplementary material available at 10.1007/s00520-024-08420-7.

## Introduction

A recent report by the World Health Organization (WHO, 2020) revealed that breast cancer is the most prevalent cancer worldwide, with 2.3 million diagnoses in 2020 and a survivor rate close to 90% in most countries (Breast cancer (who.int)). Despite the effectiveness in eradicating the disease, cancer treatments often induce many collateral effects such as fatigue [1], lymphedema [2], bone loss [3], and psychosocial impairments [4].

At the diagnosis of cancer, life of patients changes drastically, but healthful lifestyle can positively influence the survivorship trajectory [5]. Physical exercise is known to reduce the appearance of fatigue, muscle weakness, and loss of bone density which are common side effects of cancer treatment and, thus, improves physical and mental quality of life (QoL) [6]. Despite the well-documented positive effects of exercise on health, a low level of physical activity among cancer patients has been generally reported [7]. Recently, fatigue, low motivation, kinesiophobia, fear of lymphedema, and inaccessible fitness facilities are the major barriers for physical activity participation in patients with cancer [8, 9].

Among all types of physical exercise, the Dragon Boat discipline is particularly gaining ground in women with breast cancer [10]. Dragon Boat is a Chinese discipline that takes its name from typical “dragon” shape of the boat, which hosts ten couples of athletes who row to rhythm following the sound dictated by a “drummer.” Pioneering research by Dr. Harris and Dr. McKenzie showed that Dragon Boat practice elicited not only physical benefit, but also improved a number of psychosocial outcomes in breast cancer survivors [10, 11].

Dragon Boat may also positively impact body image, a psychological construct that has recently gained attention in breast cancer survivors [12]. Body image is the internal representation that individuals have of their own body and physical appearance [13]. It is a multidimensional construct comprising both positive and negative features [14, 15]. Body image may be positive (i.e., a source of satisfaction, pleasure, and well-being), and it occurs when individuals are able to accept, appreciate, and respect their body [16]. Positive body image has been related to life satisfaction and healthy behaviors [17, 18]. Regular physical exercise can promote a positive body image [19–21], as emerged in a meta-analysis showing that individuals who engage in regular physical exercise reported higher levels of positive body image than individuals who do not exercise regularly [22, 23]. Indeed, the engagement in physical exercise may improve body image by helping individuals to shift their attention from physical appearance to functional aspects of their body [24, 25]. The positive effects of exercise on body image also emerged from exercise intervention programs, as shown in a meta-analysis [22] displaying that exercise interventions (*M* length = 12.69 weeks; range, 4 to 52 weeks) improved body image compared to control conditions. Evidence suggests that 10 to 14 weeks of Dragon Boat may lead to a positive body image, higher QoL, and enhanced social support and cohesion [26–29]. However, meaningful effects on psychological dimensions may be achieved with a longer duration of the Dragon Boat activity, since psychological changes take time to be attained, especially if friendships, strong bonds, teamwork, and personal physical changes have yet to be developed. In accordance, a qualitative study on novice (i.e., first year of a dragon boating program) shows that social benefits of the Dragon Boat activity include connecting to veteran team members, who understand the breast cancer experience [30]. Most of these researches conducted on Dragon Boat have focused on predicting intentions to engage in Dragon Boat [26, 27] and have employed a qualitative methodology [30].

The present study aimed to determine how, compared to a home-based exercise program, a structured and supervised short-term Dragon Boat training can affect body composition and physical function in women who have been operated for breast cancer. Moreover, since improvements in physical function may positively impact body image by helping women to shift their focus from physical appearance to functional aspects of their body, we sought to investigate if Dragon Boat activity could promote a positive body image (i.e., body appreciation) and, in turn, physical and mental QoL.

## Materials and methods

### Participants

Thirty-two women, belonging to the same local association for women operated for breast cancer, volunteered to participate. Participants’ eligibility was determined using a clinical history questionnaire. Inclusion criteria were as follows: women ≥ 45 years, operated for breast cancer, and with a body mass index (BMI) range between 18 and 30 kg·m^−2^. Exclusion criteria were as follows: history of any other co-morbidity or chronic diseases and regular participation to structured physical exercise programs (> 2 weekly sessions of moderate or vigorous exercise). All the participants presented a diagnosis of breast cancer, with consequent removal surgery and adherence to treatment. None of the participants had ever practiced Dragon Boat or any rowing activity before. A total of thirty-one women completed the study, whose characteristics are reported in Table 1.

**Table 1:** Participants’ characteristics at baseline

	DB (*N* = 18)	HG (*N* = 13)	Difference at baseline
Age (years)	56.40 ± 7.29	59.90 ± 9.67	*p* = .102
Height (cm)	159.53 ± 6.28	156.50 ± 5.95	*p* = .242
Body mass (kg)	66.97 ± 17.38	72.10 ± 16.59	*p* = .388
BMI (kg·m^−2^)	26.78 ± 6.37	29.40 ± 6.60	*p* = .263
Fat mass (%)	32.26 ± 9.05	35.79 ± 9.29	*p* = .423
Time since diagnosis (years)	7.33 ± 3.77	10.07 ± 7.38	*p* = .186
Stage of cancer at diagnosis	Stage 0: 0% Stage 1: 6.7% Stage 2: 40.0% Stage 3: 40.0% Stage 4: 0% Unknown: 13.3%	Stage 0: 7.7% Stage 1: 15.4% Stage 2: 15.4% Stage 3: 15.4% Stage 4: 7.7% Unknown: 38.5%	*p* = .114
Type of surgery	Mastectomy: 60.0% Quadrantectomy: 40.0%	Mastectomy: 73.3% Quadrantectomy: 16.6%	*p* = .187
History of lymphedema	No: 64.3% Yes: 35.7%	No: 53.8% Yes: 46.2%	*p* = .581
Adjuvant therapy	Chemotherapy: 73.3% Radiotherapy: 6.7% Tamoxifen: 20.0% Calcium/Vitamin D: 0.0% Estrogen suppressor: 0.0%	Chemotherapy: 61.5% Radiotherapy: 7.7% Tamoxifen: 0.0% Calcium/Vitamin D: 23.1% Estrogen suppressor: 7.7%	*p* = .118

Data are presented as means ± SD. *BMI* body mass index, *DB* Dragon Boat group, *HG* home-based non-supervised training program group

### Study design and procedures

The study was a parallel randomized trial. After signing a written consent form, eligible participants were randomly assigned to the Dragon Boat (DB) exercise program or to a home-based non-supervised training program (HG).

During the first visit, participants answered to a medical questionnaire and completed the self-reported questionnaires. Subsequently, the participants underwent the following measurements: body composition analysis and upper limb circumferences, handgrip tests, 30-s chair stand test (30CST), active shoulders mobility (scratch test, arm flexion, and abduction), and the 6-min walking test (6MWT).

Total body composition was measured via bioelectrical impedance analysis (BIA, BIA 101 BIVA® PRO, Akern, Florence, Italy). Using the same phase-sensitive single-frequency BIA but adding 4 extra electrodes (on both hands and feet) for regional body composition analysis, upper body lean soft tissue and phase angle were obtained. The upper limb circumference of both arms was measured with an anthropometric tape (SECA, Genova, Italy) by the same operator. The measure was obtained in the middle point between the olecranon and the acromial process, with the arm relaxed along the side of the body, as per NHANES guidelines [31].

A digital handgrip was used to measure upper limb strength (Biometrics Ltd, Newport, UK) [32]. Participants were asked to hold the dynamometer in the hand, keeping the arm flexed to 90°. At the operator verbal signal, participants squeezed the dynamometer with maximum effort, for about 5 s. The test was repeated three times per side, and the best trial, yielding the maximum absolute value expressed in kilograms, was selected and included in the analyses.

The range of movement (ROM) tests was administrated by the same operator and was repeated three times for each arm; the best of the three attempts was considered for the analysis. The following test and procedures were adopted for both arms:

- Shoulder flexibility (Apley’s Scratch test), participants were asked to bend one elbow above and the other below the shoulders and try to have the palm touching behind their back. The distance between fingers was used to measure the ROM. The limb bended over the head indicated the side of the measurement.
- Shoulder flexion, the measure was obtained in the standing position; patients started with the palm of the hand facing the body and slowly lifted the arm on the sagittal plane bringing the hand over the head.
- Shoulder extension, from the same starting position, patients were asked to extend the arm away from the back (on the sagittal plane) keeping the neutral position and without rotating the trunk.
- Shoulder abduction, from the same starting position, patients were asked to move the humerus laterally (on the frontal plane) and away from the body, keeping the elbow extended.

For each mobility test, participants were asked to reach the designed position without pain, after checking and eventually correct for any postural compensation; using the greater tubercle (flexion and extension) or the acromial process (abduction) as the fulcrum, a goniometer (SECA, Genova, Italy) was used to record the joint excursion [33].

Afterwards, participants underwent to a strength test for the lower limbs (30CST) [34]. The participant started seated in the middle of a chair and was asked to sit and stand with the arm crossed on the chest, as many times possible in 30 s.

The visit ended with an indirect test to evaluate cardiorespiratory fitness (6-min walking test, 6MWT) [35]. The 6MWT was performed asking the participants to walk on a rectangle of 8 × 9 m. To compare the performance during the test with the healthy population, the Enright-Sherill equation for women was used to predict the 6MWT distance based on height, weight, and age of each participant [36]. The results of each test were then compared with the expected distance. The ONCORE equation was instead used to estimate VO2peak using the 30CST repetitions and body mass as per Díaz‑Balboa [37].

All tests were repeated in the same order and at the same time of the day ~ 3 to 5 days after the last training session.

The study was approved by the Human Ethical Commission of the Department of Biomedical Sciences (HEC-DSB 08/2021) of the University of Padova, in accordance with Helsinki’s declaration of 1995 as modified in 2000.

### Self-report questionnaires

#### Body Appreciation Scale-2 (BAS-2 [18, 38])

BAS-2 is a self-report measure assessing acceptance of one’s body, respect and care for one’s body, and protection of one’s body from unrealistic beauty standards. All items were rated on a 5-point scale (1 = *never*, 5 = *always*) and an overall score was computed as the mean of all items, so that higher scores reflect greater body appreciation. Scores on the Italian version of the BAS-2 have been shown to reduce to a 1-dimensional factor and to have adequate internal consistency and construct validity [38].

#### Short Form Health Survey-12 (SF-12 [39])

SF-12 is a brief version of the SF-36 health survey. The SF-12 evaluates eight dimensions related to an individual’s life that can be influenced by the presence of a disease. Answers can be provided through dichotomous yes/no answers or through items on a 3- or a 5-point Likert scale. In addition to the eight dimensions, the SF-12 produces two summary scores evaluating physical and mental health. Higher scores of the questionnaires are associated with higher physical and mental QoL. The Italian version of the SF-12 showed good content, construct, and criterion validity [2]. For the purposes of the study, we considered only the Physical Component Score (PCS) and the Mental Component Score (MCS).

### Training protocol

Participants were engaged in a 12-week intervention, which involved 3 trainings per week. All participants trained together on the same days. Each DB training lasted ~ 60 min and consisted for the initial 4 weeks of half an hour of training on the ground and only the following half of the session in the river water; subsequently, from week 4 to 8, participants were trained for 20 min on land and 40 min on the Dragon Boat; lastly, from week 8 to 12, participants underwent only 10–15 min on land (warm-up) and then almost an hour on the boat. This allowed everyone to approach the discipline gradually, without incurring injuries or discomfort.

The warm-up on the ground in the first period also included a more demanding strength workout with basic and multi-joint exercises, performed with free body, or with slight overload, aimed at strengthening the participants. With the continuation of the project, the dry work only provided for a general warm-up.

The HG program consisted of ten exercises to be performed for 60 s each, which could be repeated several times in accordance with the participant feelings. The exercises were explained through short explanatory videos. Participants were also asked to log the exercises completed and the date of training in a registration form. The comprehensive training programs for both groups are detailed in the Supplementary File.

### Statistical analysis

Statistical analyses were conducted using IBM SPSS statistics, version 28.0. An independent samples *t*-test was used to test baseline differences between groups. A 2-way repeated measure analysis of variance (ANOVA) was performed (time, within-subject factor; group, between-subject factor) in order to assess between-group differences following the intervention. When the ANOVA *F* test was significant, multiple comparisons (post hoc analyses) were corrected with Bonferroni correcting factor.

With respect to positive body image and QoL, the reliable change (i.e., the extent to which a change after intervention is reliable [40]) was evaluated. The use of reliable change allows to examine individual differences in changes over time, instead of only looking at average changes in a sample as a whole. To investigate the reliable change of each participant of the DB intervention, the Reliable Change Index (RCI, [40]) was calculated. The RCI requires (1) estimates of a scale’s internal consistency and (2) standard deviation for a given population. The threshold for reliable change is calculated as 1.96 times the standard error of the difference between scores of a measure administered on two occasions (pre- and post-training). Following Jacobson and Truax (1991) approach, the standard error of measurement (${S}_{E}$) was first calculated using:

$${S}_{E}={S}_{1}\sqrt{1-{r}_{xx}}$$

where ${S}_{1}$ is the standard deviation at pre-test and ${r}_{xx}$ is the internal consistency of the measure, and the standard error of the difference score (${S}_{diff}$) is derived as follows:

$${S}_{diff}=\sqrt{2{\left({S}_{E}\right)}^{2}}$$

Finally, RCI was calculated as follows:

$$RCI=\frac{{X}_{2}-{X}_{1}}{{S}_{diff}}$$

where ${X}_{2}$ is the individual post-test and ${X}_{1}$ is the individual pre-test.

The RCI was calculated for each participant of the DB and HG groups from T0 (pre-training) to T1 (post-training).

## Results

### Attendance rate

On average, in the DB group, we observed a participation of 20.33 ± 7.98 sessions corresponding to ~ 60% of planned training (35 sessions). In particular, 9 out of 18 participants completed ≥ 65% of the training sessions; 4 participants completed ≥ 80% of the sessions (28/35 sessions).

In contrast, only one participant out of 13 in the HG reported to have completed the circuit training for 13 days, and another one declared to have not adhered to the suggested training program. All the other participants in the HG sporadically completed the suggested training program.

None of the participants reported discomfort, pain, or injury caused by DB activity practice; the absence from training was in fact mainly justified by other personal commitments. Similarly, participants in the HG did not self-report any physical problem that could have affected their participation to the proposed exercise program.

### Body composition

Body composition and upper limb circumferences did not change following the 12-week DB intervention (*p* > 0.05 in all cases, Table 2). Moreover, we did not observe any significant difference between the operated and non-operated arm.

**Table 2:** Results from body composition analysis

	DB (*N* = 18)		HG (*N* = 13)		*t*_(29)_ baseline	Time*group
	Pre	Post	Pre	Post		
Body mass (kg)	67.64 ± 16.72	67.39 ± 17.48	72.85 ± 15.76	73.08 ± 16.23	.388	.450
Lean soft tissue (kg)	44.54 ± 5.48	44.37 ± 5.98	45.74 ± 4.58	45.92 ± 5.21	.527	.394
Fat mass (kg)	23.68 ± 11.42	23.02 ± 11.92	27.11 ± 11.87	27.15 ± 11.98	.423	.364
Total body water (L)	32.73 ± 4.103	32.62 ± 4.49	33.79 ± 3.61	33.93 ± 4.22	.459	.487
ECW (L)	16.44 ± 1.74	16.52 ± 1.88	17.31 ± 1.97	17.02 ± 2.40	.204	.395
ICW (L)	16.29 ± 2.70	16.11 ± 2.80	16.48 ± 2.02	16.91 ± 2. 23	.445	.164
Phase angle (°)	5.09 ± 0.53	5.00 ± 0.47	4.94 ± 0.47	5.15 ± 0.57	.418	.151
Operated limb						
Lean soft tissue (kg)	1.93 ± 0.41	2.07 ± 0.39	2.17 ± 0.29	2.14 ± 0.26	.084	.154
Phase angle (°)	4.51 ± 0.60	4.45 ± 0.60	4.62 ± 0.37	4.96 ± 1.22	.582	.201
Circumference (cm)	29.36 ± 5.13	29.41 ± 5.16	30.62 ± 5.08	31.02 ± 5.24	.505	.583
Non-operated limb						
Lean soft tissue (kg)	1.73 ± 0.89	2.02 ± 0.44	2.13 ± 0.22	2.10 ± 0.44	.131	.115
Phase angle (°)	4.66 ± 0.54	4.62 ± 0.66	4.71 ± 0.46	4.69 ± 0.53	.802	.849
Circumference (cm)	29.50 ± 5.07	28.56 ± 5.64	30.54 ± 4.89	30.57 ± 3.91	.572	.313

Data are mean ± SD. *ECW* extracellular water, *ICW* intracellular water, *DB* Dragon Boat group, *HG* home-based non-supervised training program group

### Physical performance tests

There were significant main effects for activity (*F*_(1,29__)_ = 14.928, *p* < 0.001, ω2 = 0.188) and a statistically significant interaction between the time and group on the 6MWT (*F*_(1,29)_ = 7.326, *p* = 0.011, ω2 = 0.017). Post hoc comparisons for group factor showed that the DB group had a better performance at baseline compared to HG (*t*_(1,29)_ =  − 2.92, *p* = 0.037) but also significantly improved during the training (*t*_(1,29)_ =  − 3.53, *p* = 0.008). As such, the difference at post-training resulted in a significantly better performance of the DB group compared to HG (*t*_(1,29)_ =  − 4.48, *p* < 0.001, Table 3).

**Table 3:** Results from physical performance tests

	DB (*N* = 18)		HG (*N* = 13)		*t*_(29)_ baseline	Time*group
	Pre	Post	Pre	Post		
6-min walk test (s)	569.28 ± 54.59	602.33 ± 52.91*	496.00 ± 76.86#	489.92 ± 94.23#	.004	.011
6-min walk test, > estimated normal (%)	72.22% (13/18)	94.44% (17/18)	76.92% (10/13)	69.23% (9/13)	-	-
30-s chair stand test (rises)	15.06 ± 2.94	19.28 ± 5.00*	11.78 ± 2.89#	12.15 ± 3.24#	.004	.011
Estimated VO2peak with ONCORE equation (ml/kg/min)	19.22 ± 2.32	20.71 ± 2.68*	17.41 ± 2.55	17.52 ± 2.89#	.050	.012
Operated limb						
Handgrip (kg)	27.05 ± 4.35	26.85 ± 4.52	25.37 ± 3.80	25.28 ± 3.35	.272	.871
Stretch test (cm)	6.83 ± 11.11	4.94 ± 8.32	8.19 ± 8.14	3.50 ± 10.73	.711	.257
Flex test (°)	172.22 ± 10.60	171.56 ± 16.27	168.85 ± 14.17	177.08 ± 34.99	.453	.367
Abduction test (°)	169.83 ± 14.10	166.67 ± 23.43	166.15 ± 16.98	168.46 ± 29.01	.515	.681
Non-operated limb						
Handgrip (kg)	28.05 ± 5.74	28.39 ± 5.52	25.38 ± 4.44	25.47 ± 4.99	.151	.817
Stretch test (cm)	10.06 ± 21.73	5.33 ± 10.31	15.23 ± 12.40	11.08 ± 12.14	.447	.922
Flex test (°)	173.44 ± 11.25	169.83 ± 20.51	168.46 ± 10.08	177.69 ± 34.13	.214	.200
Abduction test (°)	161.11 ± 41.54	164.56 ± 28.43	168.46 ± 15.73	171.00 ± 28.73	.550	.930

Data are mean ± SD. *statistically different from pre value (*p* < .05); #statistically different from DB group (*p* < .05). ONCORE equation was derived from repetitions of the 30-s chair stand test as per Díaz‑Balboa 2022 [16]. *DB* Dragon Boat group, *HG* home-based non-supervised training program group

The 30CST showed a significant main effect for group (*F*_(1,29)_ = 20.470, *p* < . 001, ω2 = 0.345), time (*F*_(1,29)_ = 10.602, *p* = 0.003, ω2 = 0.081), and a statistically significant interaction between time and group (*F*_(1,29)_ = 7.357, *p* = 0.011, ω2 = 0.055). Post hoc comparisons showed that the DB group significantly improved during training (*t*_(1,29)_ =  − 4.61, *p* < 0.001). As such, the difference at post-training resulted in a significantly better performance of the DB group compared to the HG (*t*_(1,29)_ =  − 5.28, *p* < 0.001). When the repetition from the 30CST was used in the ONCORE equation to estimate the aerobic capacity, significant main effects for group (*F*_(1,29)_ = 7.552, *p* = 0.010, ω2 = 0.098), time (*F*_(1,29)_ = 9.503, *p* = 0.004, ω2 = 0.021), and a statistically significant interaction between time and group (*F*_(1,29)_ = 7.193, *p* = 0.012, ω2 = 0.015) emerged. Post hoc comparisons showed that the DB group significantly improved during training (*t*_(1,29)_ =  − 4.45, *p* < 0.001). As such, the difference at post training resulted in a significantly better status of the DB group compared to HG (*t*_(1,29)_ =  − 3.38, *p* = 0.011).

No other significant differences were found for any of the mobility or for handgrip tests. Moreover, any differences between the operated and non-operated arm were found at baseline or in response to the interventions.

### Self-report questionnaires

The DB and HG groups did not differ in terms of body appreciation and physical and mental QoL at baseline (pre-training, Table 4). Furthermore, the 2-way ANOVA revealed no significant between-group differences following the intervention with respect to body appreciation and physical and mental QoL (Table 4). Specifically, pertaining to the BAS-2, no significant effects for time (*F*_(1,27)_ = 0.524, *p* = 0.476, ω2 = 0.020), group (*F*_(1,27)_ = 0.361, *p* = 0.553, ω2 = 0.014), and interaction (*F*_(1,27)_ = 0.799, *p* = 0.380, ω2 = 0.030) emerged. The same results apply for the SF-12 mental and physical QoL: time (respectively, *F*_(1,27)_ = 1.646, *p* = 0.213, ω2 = 0.070 and *F*_(1,27)_ = 1.477, *p* = 0.237, ω2 = 0.063), group (respectively,* F*_(1,27)_ = 0.017, *p* = 0.896, ω2 = 0.001 and *F*_(1,27)_ = 2.581, *p* = 0.122, ω2 = 0.105), and interaction (respectively,* F*_(1,27)_ = 0.176, *p* = 0.679 ω2 = 0.008 and *F*_(1,27)_ = 0.059, *p* = 0.810, ω2 = 0.003) were non-significant.

**Table 4:** Self-report questionnaire results

	DB (*N* = 16)		HG (*N* = 13)		t_(27)_ baseline	Time*group
	Pre	Post	Pre	Post		
BAS-2	3.83 ± .76	3.97 ± .46	3.76 ± .83	3.75 ± .66	.649	.380
SF-12 Mental QoL	46.44 ± 8.71	49.13 ± 7.36	47.55 ± 10.79	48.92 ± 9.54	.992	.68
SF-12 Physical QoL	46.89 ± 7.11	44.89 ± 9.14	41.31 ± 10.39	39.98 ± 6.73	.380	.81

*DB* Dragon Boat group, *HG* home-based non-supervised training program group, *BAS-2* Body Appreciation Scale-2, SF-12 Short Form Health Survey-12, QoL quality of life

### Reliable change

Self-report questionnaires (i.e., SF-12, BAS-2) were completed by 16/18 participants of the DB group. Among these 16 participants, participant number 1 did not complete the SF-12 pre-DB training and participant number 13 did not complete the BAS-2 pre-DB training, allowing us to compute the RCI for a total of 15 participants. As regards the BAS-2 and the mental health score of the SF-12, 20% (3/15) of participants obtained a reliable change from pre- to post-DB training (i.e., body appreciation and mental QoL improvements). No reliable changes emerged for the physical health score of the SF-12 (Table 5).

**Table 5:** Reliable Change Index (RCI) for the DB group

Participant	BAS-2	SF-12 Mental QoL	SF-12 Physical QoL
1	**2.86**	-	-
2	0.00	**3.24**	− 0.90
3	0.00	0.00	0.00
4	1.07	0.45	− 0.36
5	− 0.36	− 1.30	− 1.89
6	0.71	0.84	− 0.68
7	**2.50**	1.21	− 0.17
8	− 1.79	**2.42**	− 1.39
9	**3.21**	**2.54**	1.72
10	− 1.43	0.27	− 2.07
11	0.00	0.79	0.42
12	− 1.43	− 2.47	− 0.53
13	-	− 0.95	0.14
14	1.07	− 0.15	− 0.27
15	0.71	0.86	1.20
16	0.71	0.45	− 0.58

*BAS-2* Body Appreciation Scale-2, *SF-12* Short Form Health Survey-12, QoL quality of life,—absence of pre-training self-report scores. Data in bold highlight individuals with a reliable change from pre- to post-DB training (threshold for reliable change > 1.96)

With respect to the HG group, self-report questionnaires (i.e., SF-12, BAS-2) were completed by all the participants (13/13). Specifically, as regards the BAS-2, all the 13 women completed the questionnaire at both time points (pre- and post- home-based non-supervised training), while only 9 participants completed the SF-12 at both time points: participant numbers 3, 4, 10, and 13 did not complete the SF-12 at pre-training. As regards the BAS-2, 15.38% (2/13) of participants obtained a positive reliable change (i.e., body appreciation improvements) from pre- to post- home-based non-supervised training, while 15.38% (2/13) of participants obtained a negative reliable change (i.e., reduction of body appreciation) from pre- to post- home-based non-supervised training. Pertaining to the mental health score of the SF-12, 1/9 participants obtained a negative reliable change (i.e., reduction of mental QoL) from pre- to post- home-based non-supervised training. No reliable changes emerged for the physical health score of the SF-12 (Table 6**)**.

**Table 6:** Reliable Change Index (RCI) for the HG group

Participant	BAS-2	SF-12 Mental QoL	SF-12 Physical QoL
1	0.00	1.86	0.07
2	0.65	1.65	0.11
3	− 0.65	-	-
4	**3.55**	-	-
5	− 0.65	− 0.12	1.85
6	− 0.32	0.16	0.18
7	− **2.90**	− **2.07**	− 0.19
8	− 0.97	− 0.03	− 1.28
9	− **2.58**	1.19	− 0.15
10	**2.26**	-	-
11	0.65	0.51	− 1.18
12	− 0.32	− 1.14	− 0.86
13	0.65	-	-

*BAS-2* Body Appreciation Scale-2, *SF-12* Short Form Health Survey-12, QoL quality of life,—absence of pre-training self-report scores. Data in bold highlight individuals with a reliable change from pre- to post- home-based non-supervised training (threshold for reliable change < / > 1.96)

## Discussion

The aim of the present study was to evaluate the effects of a short-term Dragon Boat program on body composition, physical functions, positive body image, and mental and physical QoL in women operated for breast cancer compared to a home-based non-supervised training program. We found that the intervention was safe and well tolerated by the participants, and that functional capacity and lower limb strength were significantly improved compared to the home exercise group.

In our study, although the 35.7% of the participants of the DB group had history of lymphedema, we had no report of related adverse events during the training period. Indeed, affected upper limb circumference remained unaltered after the intervention in both DB and HG, indicating no swollen tissue. Despite Koehler and colleagues [41] have recently observed that women with lymphedema have limited function and participation to Dragon Boat activities, we have not observed any particular restriction between women with or without history of lymphedema. It is possible that the presence of lymphedema could be more limiting at higher level of physical demands, such as during competition, with respect to the first learning period as per our study.

From a functional standpoint, surprisingly we did not observe any improvement in terms of upper limb strength or ROM. Strength was measured via handgrip, which is not a specific test for the activity performed but correlates with several health outcomes in breast cancer survivors [32]. One of the possible side effects of breast cancer surgery is a decrease in grip strength, due to the incisions on the major structures involved in the grip movements [42]; Cantarero-Villanueva reported a median of 18.3-kg handgrip strength in women after the first 6 months from initial therapy [32]. More recently, Esteban-Simón reported an average of 25.9 kg in the handgrip test in women who completed the primary treatments of the disease within ~ 4.5 years since the diagnosis [43], showing a trend of strength recovery during the post-operative phase. At baseline, the participants in our study had similar levels of strength and time since diagnosis to Esteban-Simón study [43], indicating a discrete level of baseline strength. This, together with the short duration of training may justify the lack of significant improvements. In this respect, another study involving 8 weeks of training failed to observe a significant increase in upper limb strength in cancer survivors [44], while when Dragon Boat was combined with resistance training for 20 weeks, an increase of 20% of upper limb strength was detected [45]. With respect to the shoulder ROM, it has to be noted that all the participants had an optimal mobility at the initiation of the intervention. This unexpected outcome can be explained by the fact that all the participants followed a physiotherapy program during the post-operative treatment.

The effect of DB training emerged in the 6MWT and in the 30CST. Both these parameters are strongly correlated with QoL in several diseases, including breast cancer [34, 35]. Moreover, it has been recently suggested that both tests could be used in women with breast cancer to estimate cardiorespiratory fitness, which is a crucial and modifiable parameter to prevent premature mortality [46]. Although specific normative values do not yet exist for breast cancer patients, a recent meta-analysis comparing different studies on this specific population of patients from all over the world estimated that a reasonable cut-off for the 6MWT could be set at 477 m [47]. Our participants had a baseline average of 539 m (range, 368–663 m); however, the subdivision in two groups highlighted a significant difference between them, with the DB gathering to most fit and 4/13 participants under the abovementioned cut-off allocated in the HG. Despite this, only the DB participants improved their performance (from 569 to 602 m, on average) falling in the normative range for healthy subjects which is 400–700 m [48, 49]. In addition, for each participant, we used the Enright and Sherrill formula [36] to predict the distance expected in a healthy women with similar age and body size. Through this comparison, we observed that 28% (5/18) of the women in the DB group were initially under the predicted value pre-training, and only 1 participant remained under the expected outcome post-training, while in the HG, the under-expected performance passed from 3 to 4. It is also important to consider that the difference in the starting levels of physical fitness may have influenced the impact of the intervention. Indeed, it is recognized that lower levels of physical fitness may necessitate longer periods of training to induce appropriate physiological adaptations [50] and greater level of motivation to engage regularly in physical activity [51]. Therefore, it is conceivable that home-based exercise may have not been the optimal choice for these individuals.

Similarly, we observed a significant improvement in the 30CST, which highlights an increase in lower body strength after DB intervention, probably due to the preparatory training that accompanied the specific activity on the boat but also thanks to the rowing itself which is a whole body activity where both upper and lower limb strength play a determinant role [52]. Indeed, although Dragon Boat is predominantly focused on the upper limbs, the lower limbs play the crucial role of providing stability and support, maintaining a stable position on the seat, and allowing rowers to apply force effectively during the paddle stroke. Moreover, Diaz-Balboa has recently proposed the 30CST as an alternative test to estimate peak oxygen uptake in breast cancer survivors [37]; using their equation, we found that 11/18 participants in the DB group had a low level of fitness and that with the proposed intervention 7/18 improved to medium or high level. These results suggest that Dragon Boat could be a useful modality to improve cardiorespiratory fitness in breast cancer survivors.

In terms of positive body image and mental and physical QoL, statistical analysis failed to highlight any between-group differences. These findings might be partly explained by the relatively small sample size. The RCI, which investigates individual differences in changes over time, highlighted reliable changes in body appreciation in some of the women (~ 20%) of the DB group, compared to a lower proportion of participants (~ 15%) of the HG group. The small amount of improvement on body appreciation may be due to the short duration of the Dragon Boat training: body image changes may take time to be attained, especially in women operated for breast cancer. Alternatively, a ceiling effect could be discussed, since a great proportion of participants of the DB group selected the best option response with respect to body appreciation (e.g., reporting “always” for love and respect towards the body) at pre-training. When significant ceiling effects are present, they can limit the responsiveness of a scale and blunt its sensitivity to change. This effect may have impacted our ability to detect improvements in body appreciation after the Dragon Boat training. In terms of QoL, DB training appeared superior compared to the home-based training program, since 20% of DB participants improved their mental QoL, whereas none of the HG participants had any improvement, with one participant having even reductions of mental QoL. This result might be explained by the absence or only minimal engagement in the home-based non-supervised training program. A recent meta-analysis observed that involvement of professionals from different disciplines, supervision, availability of feedback, and progress information are important key factors associated with adherence to exercise program [53]. All these aspects can be guaranteed during group-in presence activity, while were lacking in the home-based exercise group, which might also explain the greater impact of the DB program.

In general, our findings support the efficacy of the Dragon Boat training in improving some aspects of well-being in breast cancer survivors and its superiority compared to a home-based non-supervised training program, providing initial support for the use of such training in this population. The absence of reliable changes in physical QoL was somehow unexpected but can be ascribed to the short duration of the training, as for many physical function outcomes.

This study has some limitations that need to be highlighted. Firstly, some of the participants were unable to engage in a structured program due to personal issues, and as such were allocated into the HG. Moreover, the two groups differed in terms of starting level of physical fitness. These two aspects could partly justify why, despite being given a home-based training program, the HG did not complete all the prescribed training sessions. Secondly, unfortunately, not all subjects completed the psychological questionnaires, which might have impacted the analysis. Moreover, the study was conducted from May to September 2021, after the second lockdown for COVID-19 in Italy. Stress and different lifestyles caused by the pandemic and related measures to restrict the transmission of the virus could have affected the results of the study. Given that studies have shown that COVID-19 had a detrimental effect on body image and QoL [54], it is difficult to quantify how the COVID-19 pandemic may have impacted our results.

## Conclusions

The present study showed that Dragon Boat activity is a feasible and effective type of training in women operated for breast cancer who have never practiced this discipline before. We observed an improvement in lower limb strength and cardiorespiratory fitness, which is positively associated with life expectancy. Moreover, we found reliable improvements in body appreciation and mental QoL in some of the women assigned to the Dragon Boat activity, which may lead individuals to be more prone to engage in healthy behaviors (e.g., adherence to treatment). We confirmed that Dragon Boat is a safe and approachable activity in women operated for breast cancer. Based on our findings, we could suggest that a longer period of Dragon Boat training would be necessary to improve also other health-related dimensions such as body composition, upper limb strength, and physical QoL.

### Supplementary Information

Below is the link to the electronic supplementary material.

Supplementary file1 (DOCX 16.5 KB)

## Data Availability

The data that support the findings of this study are available from the corresponding author upon reasonable request.
