# Supplementary material for: Keep calm and keep rowing: the psychophysical effects of dragon boat program in breast cancer survivors
Source: Support Care Cancer. 2024 Mar 8;32(4):218. doi: 10.1007/s00520-024-08420-7 (PMC10924022; doi:10.1007/s00520-024-08420-7)
Supplement: Supplementary file 1 — Supplementary file1 (DOCX 16.5 KB) [file 520_2024_8420_MOESM1_ESM.docx]

Supplementary File: TRAINING PROGRAMS

**Dragon Boat Training**

The 12-week program was divided in 3 main phases with different primary goal:

| **Weeks** | **Phase** | **Goal** | **Training session** |
| --- | --- | --- | --- |
| 0-4 | General | - Improve endurance and strength - Learning the rowing technique | - 15 min of warm up (walking, mobilization exercise) - 15 min of total body circuit training (squat, rowing with TheraBand, later rise, crunches, lunges, biceps curl) - 30 min on the Boat (3x10 min of continuative rowing) |
| 5-8 | Base | - Increase aerobic capacity and strength - Implementing the rowing technique - Increase intensity on the Boat - Complete at least 45 minutes on the boat | - 10 min of warm up (walking, mobilization exercise) - 10 min of total body circuit training - 30-45 min on the Boat   - interval training (i.e. 3min regular rowing + 30 sec high intensity rowing + 30 sec very low intensity rowing + 3 min rest)   - increasing intensity (i.e. increasing by 10 paddles/min every 2 minutes) |
| 9-12 | Specific | - Increase aerobic capacity and strength - Optimizing the rowing technique - Increase volume and intensity on the Boat - Complete at least 60 minutes on the boat | - 15-20 min of warm up with circuit training - 50-60 min on the Boat   - interval training (i.e. 3min low rowing + 1 min high intensity rowing + 30 sec very low intensity rowing + 3 min rest)   - increasing intensity (i.e. increasing by 10 paddles/min every 2 minutes) |

**Home-Based Training Program**

Following 15 min of warm up (walking, mobilization exercise), participants were invited to perform the following program based on 10 exercises:

1. Squat
2. Rowing with elastic band
3. Lateral raises
4. Crunches
5. Shoulder press
6. Lunges (one side)
7. Lunges (other side)
8. Biceps curl
9. Crunches
10. Plank

Each exercise had to be done for 30 seconds. Intensity was increased by allowing at the beginning a rest in the between and the following instruction were given:

- 15 second of exercise
- 10 seconds of rest
- 15 seconds of exercise
- 20 second of rest before passing to the next exercise

Intensity increased when women were able to perform the same exercise for 30 consecutive seconds before passing to the next exercise.

Volume distribution of the training was planned to increase upon each participants ability; the instructions were to start with 2 round of the circuit (20 min of training) and try to increase to 3 times when all exercise were tolerated for 30 consecutive seconds (30 min of training).
